# Association between *NOTCH3* gene and Parkinson’s disease based on whole-exome sequencing

**DOI:** 10.3389/fnagi.2022.995330

**Published:** 2022-12-09

**Authors:** Qian Zeng, Hongxu Pan, Yuwen Zhao, Yige Wang, Qian Xu, Jieqiong Tan, Xinxiang Yan, Jinchen Li, Beisha Tang, Jifeng Guo

**Affiliations:** ^1^Department of Neurology, Xiangya Hospital, Central South University, Changsha, China; ^2^Key Laboratory of Hunan Province in Neurodegenerative Disorders, Central South University, Changsha, China; ^3^Bioinformatics Center & National Clinical Research Center for Geriatric Disorders, Xiangya Hospital, Central South University, Changsha, China; ^4^Center for Medical Genetics & Hunan Key Laboratory of Medical Genetics, School of Life Sciences, Central South University, Changsha, China; ^5^Department of Geriatrics, Xiangya Hospital, Central South University, Changsha, China; ^6^Hunan International Scientific and Technological Cooperation Base of Neurodegenerative and Neurogenetic Diseases, Changsha, China; ^7^Engineering Research Center of Hunan Province in Cognitive Impairment Disorders, Central South University, Changsha, China

**Keywords:** CADASIL, whole-exome sequencing, common variants, NOTCH3, Parkinson’s disease

## Abstract

**Objective:**

Cerebral autosomal dominant arteriopathy with subcortical infarcts and leukoencephalopathy (CADASIL) is a hereditary cerebral small vessel disease caused by mutations in the *NOTCH3* gene. Previous studies have established a link between *NOTCH3* variants and Parkinson’s disease (PD) in terms of neuropathology and clinical characteristics. In this study, we aimed to explore the role of *NOTCH3* gene in PD in a large Chinese cohort.

**Methods:**

A total of 1,917 patients with early-onset or familial PD and 1,652 matched controls were included. All variants were divided into common or rare types by minor allele frequency (MAF) at a threshold of 0.01 (MAF > 0.01 into common variants and others into rare variants). Common variants were subjected to single-variant tests by PLINK, then gene-based analyses were used for rare variants with the optimized sequence kernel association test (SKAT-O). For genotype–phenotype correlation assessment, regression models were conducted to compare clinical features between the studied groups.

**Results:**

Three common variants (rs1044006, rs1043997, and rs1043994) showed a nominal protective effect against PD. However, none of these SNPs survived Bonferroni correction. The results in the validation cohort revealed a significant but opposite association between these variants and PD. The gene-based analyses of rare variants showed no significant associations of *NOTCH3* with PD. Although we did not find significant associations in the following genotype–phenotype analysis, the higher clinical scores of motor symptoms in *NOTCH3*-variant carriers were of interest.

**Conclusion:**

Our results indicated that *NOTCH3* gene may not play an important role in the early-onset or familial PD of Chinese population.

## Introduction

Parkinson’s disease (PD) is the second most common neurodegenerative disease. While the etiology of PD is not fully understood, environmental factors, aging, and genetic factors are often reported as triggers. A recent study has reported an overall frequency of known PD gene mutations of 1.4% in a large United Kingdom population-based cohort (Tan et al., 2019). Interestingly, marked heritability can be observed in some PD families without known genetic factors. Using genome-wide complex trait analysis for PD, it is estimated that only 27% of PD heritability is accounted for (Keller et al., 2012). Apart from known PD genes, it is worth exploring unknown genetic factors contributing to PD.

Cerebral autosomal dominant arteriopathy with subcortical infarcts and leukoencephalopathy (CADASIL), the most common hereditary cerebral small vessel disease (CSVD), is caused by pathogenic mutations in *NOTCH3* located on chromosome 19p13 (Joutel et al., 1996). It is clinically characterized by recurrent strokes, progressive subcortical dementia, migraine and psychiatric conditions (Dichgans et al., 1998). The *NOTCH3* gene encodes a single-pass transmembrane protein in smooth muscle cells and pericytes (Joutel et al., 2000). The extracellular domain of the protein contains 34 epidermal growth factor (EGF)-like repeats. Each EGF-like repeat (EGFr) domain comprises six conserved cysteine residues that form three disulfide bonds. Virtually all CADASIL mutations lead to the loss or addition of a cysteine residue and therefore to an odd number of cysteine residues within a given EGFr domain (Joutel et al., 1997).

As far as PD is concerned, the inclusions of aggregated misfolded a-synuclein, also known as Lewy bodies, play a significant role in the PD neurodegenerative process (Marogianni et al., 2020). CADASIL shares some similarities with PD in the pathology of proteinopathies. Several studies on CADASIL have exhibited dramatic abnormal protein accumulation in the vascular media of penetrating arteries (Monet-Lepretre et al., 2013; Zhang et al., 2015). Protein accumulation includes the conformational change of NOTCH3 may result from vascular redox changes (Zhang et al., 2014). Recent work has implicated that overlapping molecular mechanisms may drive PD and CADASIL (Young et al., 2020). For example, dopamine is known to play a role in the formation of multimerization of synuclein (Planchard et al., 2014; Mor et al., 2019). Dopamine and norepinephrine can also form conjugates with the NOTCH3 N-terminal fragment, which facilitate multimerization of the protein (Young et al., 2020). In 2003, Van Gerpen et al. reported a case of parkinsonism diagnosed as CADASIL for the first time (Van Gerpen et al., 2003). A later study described a patient diagnosed as CADASIL by skin biopsy, presenting with parkinsonism (Wegner et al., 2007). More and more studies have confirmed that the parkinsonian phenotype may represent one of the CADASIL manifestations (Valenti et al., 2011; Ragno et al., 2013; Erro et al., 2014; Guo et al., 2021). A recent analysis on the magnetic resonance imaging of 139 PD patients demonstrated that rare *NOTCH3* variants might be involved in the neuropathology associated with idiopathic PD (Ramirez et al., 2020). However, the contribution of the *NOTCH3* variants to the risk and clinical characteristics of PD has not been systematically described.

The pace of genetic discovery in complex diseases has accelerated exponentially for decades. In the last dozen years, many studies have been working on the genetic risk of PD. Multiple new technologies, such as next-generation sequencing (NGS) and single-cell RNA sequencing, have emerged to help with understanding the genetic architecture of disease. Whole-exome sequencing (WES) is a powerful tool to detect genes causative of multifactorial diseases, including PD. Therefore, to evaluate the association between *NOTCH3* and PD, we performed WES in a Chinese cohort and explored the clinical features between variant carriers and non-carriers.

## Materials and methods

### Study subjects

PD patients with age at onset (AAO) no more than 50 years or with a family history of PD were recruited from Xiangya Hospital and other cooperating centers of Parkinson’s Disease & Movement Disorders Multicenter Database and Collaborative Network in China (PD-MDCNC, http://pd-mdcnc.com). The diagnoses were confirmed by experienced movement disorder specialists according to the United Kingdom Parkinson’s Disease Society Brain Bank criteria (Hughes et al., 1992) or Movement Disorders Society (MDS) clinical diagnostic criteria (Postuma et al., 2015). Patients underwent a comprehensive neurological assessment. Demographic and clinical data, including the age, AAO, disease duration, as well as motor and non-motor manifestations, were retrieved from the PD-MDCNC database. Controls of Chinese ancestry enrolled from communities had no neurological or psychiatric system diseases. Written informed consents were obtained from all participants, and this study was approved by the Ethics Committee of Xiangya Hospital (Central South University). DNA of PD patients was isolated from peripheral blood leukocytes.

### Whole-exome sequencing analysis

As described in our previous study (Zeng et al., 2022), WES was performed and quality control was conducted. The Agilent SureSelect Human All Exon V6 Kit was used for exome capture. Paired-end sequencing was generated by the Illumina X10 platform with an average coverage of 123 times. The Burrows-Wheeler Aligner (BWA) was used to align sequence reads to the human reference genome (hg19; Li and Durbin, 2009). PCR duplicates were removed with Picard tools.^1^ Filtered alignments were further processed to improve the alignment quality, including base quality-score recalibration (BQSR) and local realignment around insertions/deletions (indels) using the Genome Analysis Toolkit (GATK; McKenna et al., 2010). ANNOVAR (Yang and Wang, 2015) and VarCards (Li et al., 2018a) were used to annotate gene regions (RefSeq, hg19), amino acid alterations, functional consequences, and allele frequencies in the East Asian population [the Genome Aggregation Database (gnomAD), Exome Aggregation Consortium (ExAC) database] of genetic variation from exome sequencing data. ReVe (Li et al., 2018b) was used to predict the effects of amino acid changes on protein functional status and the threshold was set to 0.7.

PLINK 1.90 (Christopher Chang/Grail Inc; Chang et al., 2015) was used for quality control. To ensure analysis of high-fidelity variants, variants were filtered to exclude those with allele depth < 5, a read depth < 10, or genotype quality score < 20. We also removed variants with a missing rate >0.1, or variants deviated significantly (*p* < 0.0001) from Hardy–Weinberg equilibrium. Individuals with discordant sex information, a missing genotype rate >0.1, or heterozygosity more than three standard deviations from the mean were excluded. Related individuals were detected by calculating identity by descent (IBD), and those with cryptic relatedness were removed (IBD > 0.15). Principal component analysis (PCA) was performed to check for genetic ancestry and only unrelated subjects of Chinese descent were retained. Besides, subjects with pathogenic/likely pathogenic variants of PD disease-causing genes were excluded from the analysis (Zhao et al., 2020).

### Statistical analysis

We selected variants in the coding regions of *NOTCH3* based on the DNA reference sequence NM_000435 (hg19, chr19:15,271,473-15,311,716) for further analysis. All variants were divided into common or rare types by minor allele frequency (MAF). Rare variants were further categorized into synonymous, missense, loss-of-function (stop gain/loss, frameshift, and splicing mutations), damaging missense (ReVe > 0.7), and previously reported to be associated with CADASIL. We performed logistic regression for common variants (MAF > 0.01) using PLINK with case/control status as an outcome. To further explore the association between *NOTCH3* common variants and PD, we conducted a validation study using data from the Parkinson’s Disease DNA Variant browser^2^ (Kim et al., 2021). This public platform integrates sequencing data from multiple sources, and the vast majority of data are from European ancestry. Difference in the allele frequency was compared by Fisher’s exact tests.

The optimized sequence kernel association test (SKAT-O) method (Lee et al., 2012) in R software (v3.6.3) was used to analyze variants with MAF < 0.01. Age at enrollment, sex and the first five principal components of ancestry were used as covariates. Besides, variants within the EGFr domain were exclusively analyzed. Clinical characteristics were compared between two groups based on their genotype: patients with rare variants which had been previously reported and patients negative for these variants. Linear and logistic regression analyses were performed for genotype–phenotype correlations with adjustment for age at enrollment, disease duration, and sex. Value of *p* < 0.05 was defined as statistically significant after Bonferroni correction.

## Results

### Characteristics of variants identified by WES

**Figure 1:**
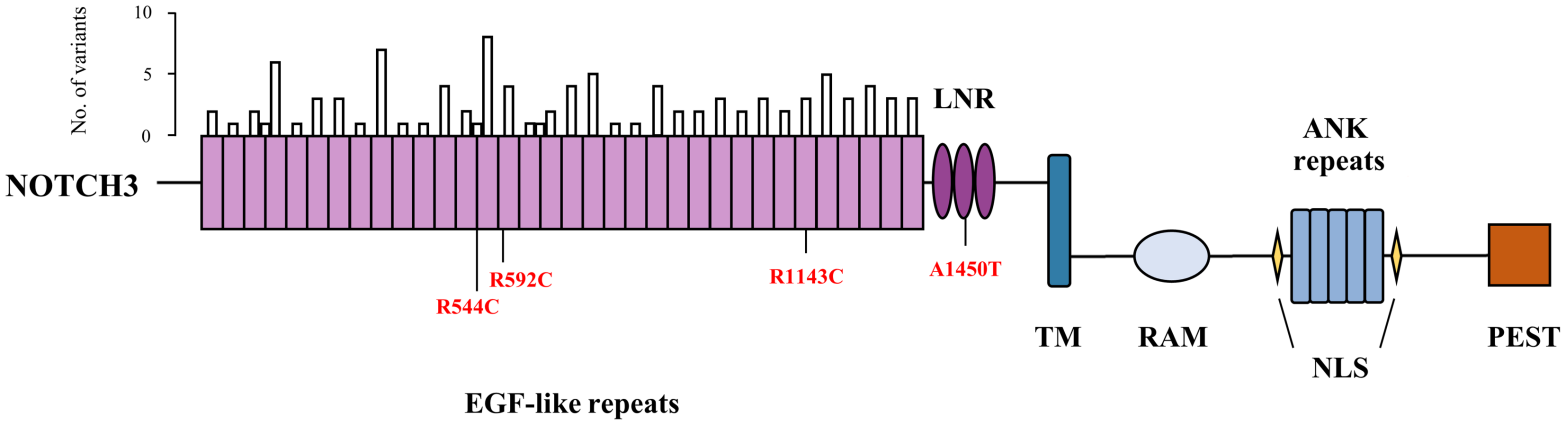
Schematic diagram of domain structure and rare variants of *NOTCH3*. The bar above represented number of rare variants within epidermal growth factor-like repeat domain. Previously reported variants were depicted as red. EGF, epidermal growth factor; LNR, Lin12-Notch repeats; TM, transmembrane domain; RAM, RBP-jκ-associated molecule domain; ANK, ankyrin; NLS, nuclear localization sequence; PEST, domain rich in proline, glutamate, serine and threonine.

All samples underwent WES and Supplementary Table 1 presented the results of the sequencing. After quality control, 1,917 patients with early-onset or familial PD and 1,652 controls were available for analysis. Brief descriptions of included samples were given in Supplementary Table 2. Among our cohort, seven common variants and 207 rare variants in *NOTCH3* gene were detected. A total of 124 nonsynonymous variants were located at coding regions (two splicing and 40 damaging missense variants). We identified 102 rare variants in the EGFr domain of *NOTCH3* in our samples, of which 24 were predicted to be damaging by ReVe. As shown in Figure 1, these rare variants were enriched in EGFr 4, EGFr 9 and EGFr 14. In addition, four variants had been reported to be associated with CADASIL, and three of them were in the EGFr domain. Further detailed information regarding the prioritized rare variants (splicing, damaging missense or previously reported) was shown in Supplementary Table 3.

### Common and rare variants association tests

Among seven common variants identified, the single nucleotide polymorphisms (SNPs) rs1044006, rs1043997 and rs1043994 showed a nominal protective effect against PD (Table 1). However, none of these SNPs survived Bonferroni correction (threshold of significance = 7.14 × 10^−3^ for seven variants). In the validation study, we found significant differences in the MAF of three SNPs between PD cases and controls when considered the whole data cohorts (Supplementary Table 4). Of note, the association direction was opposite from our study, that the altered allele was higher in PD patients in the validation cohort. In analyses restricted to each cohort separately, only rs1044006 was associated with a higher odds of PD in the International Parkinson’s Disease Genomics Consortium (IPDGC) genome-wide association study (GWAS) Cohort, and the association direction of three SNPs varied in different cohorts.

**Table 1:** Association analysis of common variants in the coding region.

Gene	Hg19 position	dbSNP ID	Ref	Alt	Case			Control			OR (95% CI)	Value of *p*
					Hom (Alt)	Het	Hom (Ref)	Hom (Alt)	Het	Hom (Ref)		
*NOTCH3*	19:15271771	rs1044009	G	A	371	905	640	333	813	499	0.90 (0.77–1.04)	0.154
	**19:15285052**	**rs1044006**	**T**	**C**	**71**	**575**	**1,269**	**64**	**549**	**1,034**	**0.79 (0.65–0.95)**	**0.014**
	**19:15292437**	**rs1043997**	**T**	**C**	**54**	**548**	**1,315**	**52**	**518**	**1,082**	**0.78 (0.64–0.95)**	**0.013**
	19:15295134	rs1043996	G	A	312	913	692	314	797	541	0.92 (0.79–1.07)	0.299
	19:15298136	rs75617410	C	A	4	130	1,783	4	151	1,497	0.82 (0.56–1.20)	0.302
	**19:15302844**	**rs1043994**	**T**	**C**	**22**	**420**	**1,475**	**40**	**418**	**1,194**	**0.74 (0.59–0.92)**	**0.008**
	19:15303225	rs3815188	G	A	278	879	760	235	754	663	1.14 (0.98–1.32)	0.102

Ref, reference allele; Alt, alteration allele; MAF, minor allele frequency; OR, odds ratio; CI, confidence interval. Bold values denote statistical significance at the *p* < 0.05 level.

To evaluate the effects of rare variants, stratified association analyses were performed, as shown in Table 2. Of the rare variants in *NOTCH3* gene, no significant difference in variant burden was found between cases and controls. Further stratification by variant properties showed similar results. In analyses restricted to variants that had been previously reported, the SKAT-O analysis revealed no significant associations between these variants and PD status (0.6% vs. 0.5%, *p* = 0.669). Considering more than 95% of CADASIL mutations are missense mutations altering the number of cysteine residues within the EGFr (Abramycheva et al., 2015), we further compared the frequencies of variants lying within the EGFr domain, and Table 2 showed a similar distribution between the two groups.

**Table 2:** Gene-based association results of rare variants.

Region	Variants group	No. of variants	Cases (*n* = 1,917)	Controls (*n* = 1,652)	Value of *p*
*NOTCH3*	All	207	300 (15.7%)	292 (17.7%)	0.804
	Synonymous	83	122 (6.4%)	112 (6.8%)	0.813
	Missense	122	177 (9.2%)	179 (10.8%)	0.599
	LoF	2	1 (0.1%)	1 (0.1%)	0.632
	Dmis	40	65 (3.4%)	72 (4.4%)	0.716
	LoF + Dmis	42	66 (3.4%)	73 (4.4%)	0.731
	Previously reported^a^	4	11 (0.6%)	8 (0.5%)	0.669
EGFr	All	102	139 (7.3%)	131 (7.9%)	1
	Synonymous	33	24 (1.3%)	31 (1.9%)	0.831
	Missense	69	115 (6.0%)	100 (6.0%)	1
	Dmis	24	43 (2.2%)	38 (2.3%)	1

LoF, loss-of-function; Dmis, damaging missense (ReVe > 0.7).

a Variants previously reported to be associated with CADASIL.

### Clinical features of PD patients with the *NOTCH3* variants

Previous studies have discovered that some CADASIL patients can present with parkinsonism (Wegner et al., 2007; Ragno et al., 2013). In our cohort, four variants had been previously reported to be associated with CADASIL. A total of 11 PD patients carrying these variants were included in our cohort. Patients that harbored at least one previously reported variant were referred to as *NOTCH3*-variant carriers, whereas those who did not were referred to as *NOTCH3*-variant non-carriers. To better understand the clinical characteristics of *NOTCH3*-variant carriers, PD patients carrying rare variants which had been previously reported were compared with non-carriers. Descriptions and results for the comparisons of the clinical features across the two groups of PD patients were included in Table 3. The sex, AAO and disease duration at evaluation of the two groups showed no statistical differences. Although the differences were not significant, we still found possible impacts of previously reported variants in *NOTCH3* on clinical severity. The results demonstrated more prevalent motor symptoms in *NOTCH3*-variant carriers, especially more severe in stiffness (*p* = 0.278), bradykinesia (*p* = 0.547) and postural instability (*p* = 0.119). With regard to dopaminergic complications, we found that dyskinesias (27.3% vs. 15.0%; OR = 2.57, *p* = 0.194) were more common in *NOTCH3*-variant carriers. In terms of non-motor features, it seemed that depression, autonomic symptom dysfunction and fatigue were more frequently observed in *NOTCH3*-variant carriers. Notably, the scores of Mini-mental state examination (MMSE) did not differ between groups.

**Table 3:** Comparison of clinical features in carriers and non-carriers of *NOTCH3* variants.

Clinical features	Previously reported variants			
	Carriers	Non-carriers	*β*/OR	Value of *p*
Demographic factors and disease characteristic				
Sex (% men)	54.5%	54.6%	1.00	0.996
Age at onset (years)	47.6 ± 8.1	46.3 ± 8.4	1.28	0.609
Age at enrollment (years)	53.4 ± 10.4	52.2 ± 9.0	1.28	0.608
Disease duration (years)	5.7 ± 5.3	5.9 ± 5.0	−0.44	0.750
Motor features				
UPDRS-Part I	3.2 ± 2.7	2.4 ± 2.0	0.78	0.197
UPDRS-Part II	12.6 ± 8.5	11.6 ± 6.7	1.02	0.566
UPDRS-Part III	29.5 ± 18.7	26.8 ± 15.7	2.58	0.552
Tremor score	2.1 ± 1.9	3.8 ± 3.7	−1.71	0.122
Stiffness score	6.8 ± 4.8	5.5 ± 4.2	1.33	0.278
Bradykinesia score	11.1 ± 7.2	9.9 ± 6.6	1.13	0.547
Postural instability score	5.2 ± 5.0	3.9 ± 3.1	1.30	0.119
Hoehn and Yahr	2.5 ± 1.3	2.1 ± 0.8	0.36	0.114
Motor phenotype			2.31	0.222^a^
PIGD dominant	72.7%	54.4%	–	–
Tremor dominant	9.1%	27.6%	–	–
Indeterminate	18.2%	18.0%	–	–
Freezing gait	9.1%	25.9%	0.25	0.205
Non-motor features				
MMSE	27.2 ± 2.1	27.1 ± 3.1	0.33	0.736
PDSS	115.9 ± 21.4	117.0 ± 28.6	−0.07	0.994
RBDQ-HK	11.2 ± 10.5	13.1 ± 16.1	−2.84	0.581
ESS	7.8 ± 7.1	7.1 ± 6.1	0.33	0.865
HRS	20.2 ± 5.8	20.1 ± 6.2	0.39	0.848
HAMD	9.1 ± 8.1	5.6 ± 5.6	3.41	0.063
PDQ39	36.7 ± 32.6	28.7 ± 25.7	6.65	0.394
PFS	49.7 ± 12.6	43.6 ± 18.9	7.65	0.317
SCOPA-AUT	9.7 ± 6.4	7.1 ± 6.6	2.43	0.297
Dopaminergic complications				
Dyskinesia	27.3%	15.0%	2.57	0.194
Wearing-off	27.3%	24.5%	1.22	0.782

OR, odds ratio; UPDRS, Unified Parkinson’s disease rating scale; PIGD, postural instability gait disorder; MMSE, Mini-mental state examination; PDSS, Parkinson’s disease sleep scale; RBDQ-HK, Rapid eyes movement sleep behavior disorder questionnaire-Hong Kong; ESS, Epworth sleepiness scale; HRS, Hyposmia rating scale; HAMD, 17-item Hamilton depression rating scale; PDQ39, The 39-item Parkinson’s disease questionnaire; PFS, Parkinson’s disease fatigue scale; SCOPA-AUT, Scales for outcomes in Parkinson’s disease-autonomic.

^a^Motor phenotypes were dichotomized into categories of PIGD vs non-PIGD.

## Discussion

The *NOTCH3* gene encodes a single-pass transmembrane protein belonging to an evolutionarily conserved NOTCH receptor family. In 1993, the data obtained from two French CADASIL families allowed the gene locus of the disease to be assigned to chromosome 19q12 (Tournier-Lasserve et al., 1993). In our study, we applied WES to a cohort of 1,917 PD patients and 1,652 controls. Genetic and phenotypic data were analyzed with regression analyses and the SKAT-O method. Our results indicated that *NOTCH3* gene may not play an important role in PD of Chinese population.

Among the common variants that were detected in our cohort, rs1044006, rs1043997, and rs1043994 exhibited suggestive associations with PD. Contrary to what we had supposed, these SNPs exerted a protective effect against PD. Furthermore, our study detected significant association results in the opposite direction in an integrated European population cohort, which can be attributed to the ethnic difference in the MAF of these SNP. But the association direction of each SNP varied when considering the genomic data sets separately. Thus, our results may be a chance finding and needs to be replicated in future studies. For rare variants, we explored the possible association of PD in different variants groups. Consistent with the findings by Dilliott et al. (2021), we did not detect a significant enrichment of rare nonsynonymous variants among PD patients when compared to the controls. To date, more than 300 *NOTCH3* mutations have been reported, and it was shown that the vast majority of the pathogenic mutations for CADASIL were clustered in EGFr 1–6 (Chabriat et al., 2020). Rare variants identified in our cohort enriched in EGFr 4, EGFr 9 and EGFr 14, and gene-based burden tests restricted to the EGFr domain also did not achieve a value of *p* <0.05. Typically, mutations in the *NOTCH3* gene are associated with CADASIL, a monogenic subtype of CSVD. In our cohort, it is unclear whether the control participants had any history of CSVD without obvious symptoms or had CSVD imaging characteristics, which could lead to false negative results.

A previous study found that parkinsonism may not be a rare manifestation in CADASIL. These patients with the R1006C mutation of *NOTCH3* had higher frequencies of akinesia, rigidity and postural instability, as well as a poor response to levodopa (Ragno et al., 2013). Recently, evidence has shown that the *NOTCH3* rare variants may significantly contribute to the increased white matter hyperintensities burden of PD patients, which in turn may negatively influence cognition (Ramirez et al., 2020). Although we did not find significant associations in the following genotype–phenotype analysis, the higher clinical scores of motor symptoms in *NOTCH3*-variant carriers were of interest, providing a possibility of the association with *NOTCH3* variants and motor signs in PD. We found stiffness, bradykinesia and postural instability were seen more commonly in PD patients with variants that had been previously reported, whereas rest tremor was always absent. Although literature data which used the Montreal Cognitive Assessment (MoCA) to assess global cognition supported an association between the presence of *NOTCH3* and cognitive impairment (Ramirez et al., 2020), we did not detect significant differences in cognitive capabilities between *NOTCH3*-variant carriers and non-carriers based on the MMSE scores.

However, this result might have occurred due to the small number of *NOTCH3*-variant carriers. Hence, it calls for a validation study that will have access to a cohort with a larger size of *NOTCH3*-variant carriers, thus increasing the power of detection. In addition, the MMSE is the most widely used cognitive screening measure for general cognitive evaluation (Anderson, 2019). Considering its variation in sensitivity and specificity, MMSE might be too insensitive to detect subtle differences between two groups.

The main strength of our study is the large sample size of Chinese origin in addition to the use of uniform clinical evaluation methods by PD-MDCNC database. However, some additional limitations need to be considered. First, the majority of non-motor symptoms were evaluated based on the patients’ responses to a questionnaire. More powerful and objective tests were needed to enhance the reliability of non-motor features. On the other hand, due to its retrospective design, the phenotypic assessment had some missing data. For future studies, missing data should be complemented to facilitate an optimal assessment. Third, because of a lack of magnetic resonance imaging, we failed to validate the relationship between white matter lesions and PD in *NOTCH3*-positive patients, which might play a role in the pathogenesis of cognitive impairment. Meanwhile, false negative results may be produced due to the absence of magnetic resonance imaging in the control participants. Finally, WES method failed to identify copy number variations and non-coding variants, so it is necessary for extensive analysis to explore the link between variants within these regions and PD.

Taken together, we comprehensively investigated the *NOTCH3* gene in a large cohort of Chinese PD patients and healthy controls. Although no evidence for significant associations between *NOTCH3* variants and PD was noted, replications in different ethnic cohorts and further functional studies are needed.

## Data availability statement

The datasets presented in this study can be found in online repositories. The names of the repository/repositories and accession number(s) can be found at: https://db.cngb.org/cnsa/, CNP0002638.

## Ethics statement

The studies involving human participants were reviewed and approved by the Ethics Committee of Xiangya Hospital (Central South University). The patients/participants provided their written informed consent to participate in this study.

## Author contributions

QZ carried out genetic analysis and wrote the manuscript. HP, YZ, and YW processed genomic data. QX, JT, XY, and JL helped the study design and data interpretation. BT and JG critically revised the manuscript. All authors contributed to the article and approved the submitted version.

## Funding

This study was supported by the Central Public-Interest Scientific Institution Basal Research Fund of Chinese Academy of Medical Sciences (grant nos. 2018-12M-HL-025 and 2019-RC-HL-025); National Natural Science Foundation of China (grant nos. 81974202, 81873785, 82071439, and U20A20355); Technology Major Project of Hunan Provincial Science and Technology Department (grant no. 2021SK1010); Hunan Province Innovative Construction Project Science (grant no. 2019SK2335); Innovative team program from Department of Science & Technology of Hunan Province (grant no. 2019RS1010); Innovation-driven Team Project from Central South University (grant no. 2020CX016); and National Key Research and Development Program of China (grant no. 2021YFC2501204).

## Conflict of interest

The authors declare that the research was conducted in the absence of any commercial or financial relationships that could be construed as a potential conflict of interest.

## Publisher’s note

All claims expressed in this article are solely those of the authors and do not necessarily represent those of their affiliated organizations, or those of the publisher, the editors and the reviewers. Any product that may be evaluated in this article, or claim that may be made by its manufacturer, is not guaranteed or endorsed by the publisher.

## Footnotes

^1^ http://broadinstitute.github.io/picard/

^2^ https://pdgenetics.shinyapps.io/VariantBrowser/
