# Supplementary material for: Association between NOTCH3 gene and Parkinson’s disease based on whole-exome sequencing
Source: Front Aging Neurosci. 2022 Dec 9;14:995330. doi: 10.3389/fnagi.2022.995330 (PMC9780269; doi:10.3389/fnagi.2022.995330)
Supplement: Supplementary file 1 [file Data_Sheet_1.docx]

Supplementary Material

# Supplementary Table 1. Summary of exome sequencing data in this study.

| **Detail information** | **Patients** | **Controls** |
| --- | --- | --- |
| *NOTCH3* region | | |
| Target region (bp) | 6,933 | 6,933 |
| Target reads | 12,299 | 12,135 |
| Target data (Mb) | 1.19 | 1.19 |
| Mean depth of target region (x) | 171.74 | 170.94 |
| Coverage of target region (%) | 100.00 | 99.56 |
| Fraction of target covered ≥ 4x (%) | 99.99 | 99.32 |
| Fraction of target covered ≥ 10x (%) | 99.73 | 98.61 |
| Fraction of target covered ≥ 30x (%) | 95.08 | 92.53 |
| EGFr region | | |
| Target region (bp) | 4,262 | 4,262 |
| Target reads | 8,553 | 8,760 |
| Target data (Mb) | 0.78 | 0.82 |
| Mean depth of target region (x) | 183.99 | 191.66 |
| Coverage of target region (%) | 100.00 | 99.43 |
| Fraction of target covered ≥ 4x (%) | 99.98 | 99.13 |
| Fraction of target covered ≥ 10x (%) | 99.62 | 98.17 |
| Fraction of target covered ≥ 30x (%) | 93.25 | 89.94 |

# Supplementary Table 2. Descriptive statistics of this study.

| **Clinical features** | **Patients**  **(n = 1,917)** | **Controls**  **(n = 1,652)** |
| --- | --- | --- |
| Sex, men (%) | 54.6% | 48.1% |
| Age at enrollment (year, mean ± SD) | 52.2±9.0 | 62.0±12.6 |
| Age at onset (year, mean ± SD) | 46.3±8.4 |  |

n, sample size; SD, standard deviation.

**Supplementary Table 3.** Prioritized rare variants identified within the *NOTCH3* gene.

| **Hg19 position** | **cDNA Alteration** | **Protein Alteration** | **dbSNP ID** | **Exon** | **Domain** | **Exonic Function** | **MAF^*^** | **Patients**  **(n = 1,917)** | **Controls**  **(n = 1,652)** | **CADD/Reve** | **Previous reported in CADASIL** |
| --- | --- | --- | --- | --- | --- | --- | --- | --- | --- | --- | --- |
| 19: 15295788 | c.2339G>A | p.C780Y | - | 15 | EGFr 20 | missense | -/- | 0/0/1917 | 0/1/1649 | 26.8/0.994 | No |
| 19: 15302669 | c.689G>A | p.G230D | - | 5 | EGFr 5 | missense | -/- | 0/1/1916 | 0/0/1652 | 26.5/0.983 | No |
| 19: 15302649 | c.709G>A | p.V237M | rs2285981 | 5 | EGFr 6 | missense | 0.0024/0.0029 | 0/0/1905 | 0/1/1638 | 23.4/0.948 | No |
| 19: 15302968 | c.482A>G | p.E161G | - | 4 | EGFr 4 | missense | -/- | 0/0/1917 | 0/1/1651 | 27.6/0.939 | No |
| 19: 15276680 | c.5585G>T | p.G1862V | - | 30 | ANK 1 | missense | -/- | 0/1/1916 | 0/0/1652 | 32.0/0.931 | No |
| 19: 15292597 | c.2582G>T | p.G861V | rs1220670491 | 17 | EGFr 22 | missense | 0/- | 0/2/1915 | 0/0/1652 | 26.3/0.923 | No |
| 19: 15289752 | c.3719G>A | p.G1240D | rs775292982 | 23 | EGFr 31 | missense | 0/- | 0/0/1917 | 0/1/1651 | 29.0/0.918 | No |
| 19: 15276251 | c.5743C>T | p.R1915C | rs752426825 | 31 | ANK 3 | missense | 0/- | 0/1/1916 | 0/0/1652 | 33.0/0.903 | No |
| 19: 15276290 | c.5704C>T | p.R1902C | rs576516079 | 31 | ANK 2-3 | missense | 0.0003/0.0006 | 0/0/1917 | 0/1/1651 | 34.0/0.897 | No |
| 19: 15289994 | c.3560G>A | p.G1187D | rs199539384 | 22 | EGFr 30 | missense | -/- | 0/2/1915 | 0/1/1651 | 25.6/0.894 | No |
| 19: 15300200 | c.1076C>T | p.P359L | rs1487017745 | 7 | EGFr 9 | missense | 0/- | 0/1/1916 | 0/0/1652 | 26.7/0.894 | No |
| 19: 15290180 | c.3455C>T | p.T1152M | rs371437217 | 21 | EGFr 29 | missense | 0.0004/0.0005 | 0/3/1914 | 0/1/1651 | 24.7/0.886 | No |
| 19: 15297982 | c.1774C>T | p.R592C | rs764148985 | 11 | EGFr 15 | missense | 0/- | 0/0/1917 | 0/1/1651 | 32.0/0.87 | Yes (Adib-Samii et al., 2010, Testi et al., 2012) |
| 19: 15289698 | c.3773G>A | p.G1258E | - | 23 | EGFr 32 | missense | -/- | 0/1/1916 | 0/0/1652 | 23.6/0.869 | No |
| 19: 15302935 | c.515G>A | p.G172D | rs775461675 | 4 | EGFr 4 | missense | 0.0002/0.0001 | 0/2/1915 | 0/6/1646 | 25.3/0.866 | No |
| 19: 15289711 | c.3760C>T | p.P1254S | - | 23 | EGFr 32 | missense | -/- | 0/0/1917 | 0/2/1650 | 24.7/0.859 | No |
| 19: 15288435 | c.4304G>C | p.R1435P | - | 24 | LNR 2 | missense | -/- | 0/1/1916 | 0/0/1628 | 23.3/0.858 | No |
| 19: 15281175 | c.5081G>A | p.R1694Q | - | 27 | - | missense | -/- | 0/0/1917 | 0/1/1651 | 32.0/0.857 | No |
| 19: 15302649 | c.709G>T | p.V237L | - | 5 | EGFr 6 | missense | -/- | 0/0/1905 | 0/1/1638 | 22.1/0.851 | No |
| 19: 15300201 | c.1075C>T | p.P359S | - | 7 | EGFr 9 | missense | -/- | 0/1/1916 | 0/0/1652 | 25.7/0.849 | No |
| 19: 15272498 | c.5941C>T | p.R1981C | rs200541224 | 33 | ANK 5 | missense | 0.00006/- | 0/1/1916 | 0/1/1651 | 28.8/0.840 | No |
| 19: 15272348 | c.6091C>T | p.R2031C | rs748196084 | 33 | - | missense | 0.0001/0 | 0/1/1916 | 0/1/1651 | 28.8/0.823 | No |
| 19: 15300137 | c.1139C>T | p.P380L | rs748814154 | 7 | EGFr 9 | missense | 0.00005/0.0001 | 0/1/1916 | 0/0/1652 | 29.4/0.817 | No |
| 19: 15292496 | c.2683C>T | p.P895S | rs774992097 | 17 | EGFr 23 | missense | 0.0002/- | 0/2/1915 | 0/0/1652 | 24.7/0.815 | No |
| 19: 15302390 | c.881C>T | p.T294M | rs146578114 | 6 | EGFr 7 | missense | 0/0 | 0/0/1917 | 0/1/1651 | 23.7/0.811 | No |
| 19: 15272501 | c.5938G>A | p.A1980T | rs367885841 | 33 | ANK 5 | missense | 0/0 | 0/1/1916 | 0/0/1652 | 27.9/0.804 | No |
| 19: 15272336 | c.6103G>T | p.G2035C | rs762666964 | 33 | - | missense | -/- | 0/1/1916 | 0/0/1652 | 22.8/0.801 | No |
| 19: 15276193 | c.5801C>T | p.A1934V | rs554059112 | 31 | ANK 3 | missense | 0.001/0.0012 | 0/4/1913 | 0/2/1650 | 27.6/0.794 | No |
| 19: 15276230 | c.5764G>T | p.V1922L | rs114813086 | 31 | ANK 3 | missense | 0.0005/0.0005 | 0/3/1914 | 0/0/1652 | 25.3/0.787 | No |
| 19: 15281185 | c.5071A>G | p.K1691E | - | 27 | - | missense | -/- | 0/1/1916 | 0/0/1652 | 23.6/0.785 | No |
| 19: 15298126 | c.1630C>T | p.R544C | rs201118034 | 11 | EGFr 13-14 | missense | 0.004/0.0036 | 0/5/1912 | 0/1/1651 | 24.9/0.782 | Yes (Hu et al., 2021, Lee et al., 2006) |
| 19: 15296335 | c.2107C>T | p.P703S | rs1243021412 | 13 | EGFr 18 | missense | 0/- | 0/1/1916 | 0/0/1652 | 24.0/0.772 | No |
| 19: 15276755 | c.5510G>A | p.R1837H | rs138265894 | 30 | - | missense | 0.0006/0.0006 | 0/0/1917 | 0/1/1651 | 29.0/0.753 | No |
| 19: 15285171 | c.4444G>A | p.G1482S | rs202085803 | 25 | LNR 3 | missense | 0/- | 0/1/1916 | 0/0/1652 | 26.1/0.753 | No |
| 19: 15298066 | c.1690G>A | p.A564T | rs374767079 | 11 | EGFr 14 | missense | 0.0013/0.001 | 0/5/1912 | 0/6/1646 | 25.6/0.735 | No |
| 19: 15281611 | c.4762A>C | p.N1588H | - | 26 | - | missense | -/- | 0/0/1915 | 0/3/1621 | 26.3/0.732 | No |
| 19: 15288391 | c.4348G>A | p.A1450T | rs201082692 | 24 | LNR 2 | missense | 0.0013/0.0028 | 0/4/1913 | 0/6/1643 | 24.1/0.732 | Yes (Yuan et al., 2019) |
| 19: 15298041 | c.1715C>T | p.P572L | rs773507679 | 11 | EGFr 14 | missense | 0.0003/0.0002 | 0/1/1916 | 0/2/1650 | 27.1/0.714 | No |
| 19: 15290208 | c.3427C>T | p.R1143C | rs60373464 | 21 | EGFr 29 | missense | 0.00005/0.0001 | 0/2/1915 | 0/0/1652 | 26.3/0.705 | Yes (Mizuta et al., 2017) |
| 19: 15296476 | c.1966G>A | p.V656M | rs560752299 | 13 | EGFr 16-17 | missense | 0/0 | 0/1/1916 | 0/0/1652 | 26.7/0.702 | No |
| 19: 15295262 | c.2411-1G>A |  | - | 16 | - | splicing | -/- | 0/0/1917 | 0/1/1651 | 25.9/0.609 | No |
| 19: 15298807 | c.1493-2->C |  | - | 10 | - | splicing | 0.0001/- | 0/1/1915 | 0/0/1650 | -/- | No |

cDNA, coding DNA; EGFr, epidermal growth factor-like repeat; LNR, Lin12/Notch repeat; ANK, ankyrin repeat; MAF, minor allele frequency; CADASIL, cerebral autosomal dominant artiopathy with subcortical infarcts and leukoencephalopathy. The genotype counts were shown as Hom/Het/Wild.

* MAF from gnomAD_exome_EAS and ExAC_EAS.

# Supplementary Table 4. Differences in the minor allele frequency of 3 common SNPs between PD cases and controls.

| **Data cohort** | **rs1044006** | | **rs1043997** | | **rs1043994** | |
| --- | --- | --- | --- | --- | --- | --- |
|  | **OR (95% CI)** | ***p* value** | **OR (95% CI)** | ***p* value** | **OR (95% CI)** | ***p* value** |
| PD Genome Project | 1.10 (0.98-1.23) | 0.107 | 1.01 (0.91-1.12) | 0.879 | 1.00 (0.90-1.12) | 0.957 |
| IPDGC Exomes | 0.97 (0.82-1.15) | 0.799 | 0.98 (0.84-1.14) | 0.823 | 1.01 (0.90-1.14) | 0.831 |
| IPDGC GWAS Cohort | 1.06 (1.00-1.11) | **0.037** | 1.03 (0.98-1.08) | 0.233 | 1.02 (0.97-1.07) | 0.439 |
| UK Biobank | 1.05 (0.64-1.64) | 0.818 | 0.93 (0.59-1.40) | 0.839 | 0.98 (0.62-1.48) | 1 |
| Total | 1.10 (1.06-1.14) | **1.027E-06** | 1.09 (1.05-1.12) | **1.836E-06** | 1.07 (1.03-1.11) | **1.500E-04** |

SNPs, single nucleotide polymorphisms; PD, Parkinson’s disease; OR, odds ratio; CI, confidence interval; IPDGC, International Parkinson's Disease Genomics Consortium; GWAS, genome-wide association study.

**References**

Adib-Samii, P., Brice, G., Martin, R. J., Markus, H. S. (2010). Clinical spectrum of CADASIL and the effect of cardiovascular risk factors on phenotype: study in 200 consecutively recruited individuals. *Stroke*.41:630-4. doi: 10.1161/STROKEAHA.109.568402.

Hu, Y., Sun, Q., Zhou, Y., Yi, F., Tang, H., Yao, L., et al. (2021). NOTCH3 Variants and Genotype-Phenotype Features in Chinese CADASIL Patients. *Front Genet*.12:705284. doi: 10.3389/fgene.2021.705284.

Lee, Y. C., Yang, A. H., Liu, H. C., Wong, W. J., Lu, Y. C., Chang, M. H., et al. (2006). Cerebral autosomal dominant arteriopathy with subcortical infarcts and leukoencephalopathy: two novel mutations in the NOTCH3 gene in Chinese. *J Neurol Sci*.246:111-5. doi: 10.1016/j.jns.2006.02.011.

Mizuta, I., Watanabe-Hosomi, A., Koizumi, T., Mukai, M., Hamano, A., Tomii, Y., et al. (2017). New diagnostic criteria for cerebral autosomal dominant arteriopathy with subcortical infarcts and leukocencephalopathy in Japan. *J Neurol Sci*.381:62-7. doi: 10.1016/j.jns.2017.08.009.

Testi, S., Malerba, G., Ferrarini, M., Ragno, M., Pradotto, L., Mauro, A., et al. (2012). Mutational and haplotype map of NOTCH3 in a cohort of Italian patients with cerebral autosomal dominant arteriopathy with subcortical infarcts and leukoencephalopathy (CADASIL). *J Neurol Sci*.319:37-41. doi: 10.1016/j.jns.2012.05.025.

Yuan, X., Li, C., Chen, X., Liu, L., Liu, G., Wen, F. (2019). A Study of Congenital Protein C Deficiency With Infancy Onset of CADASIL in a Chinese Baby. *J Pediatr Hematol Oncol*.41:e210-e5. doi: 10.1097/MPH.0000000000001436.
